# Supplementary material for: A Realist Evaluation of Team Interventions in Acute Hospital Contexts—Use of Two Case Studies to Test Initial Programme Theories
Source: Int J Environ Res Public Health. 2021 Aug 14;18(16):8604. doi: 10.3390/ijerph18168604 (PMC8393900; doi:10.3390/ijerph18168604)
Supplement: Supplementary file 1 [file ijerph-18-08604-s001.zip › ijerph-1290688-supplementary.pdf]

Supplementary file 1

Composition and expertise of content expert advisory panel that participated in theory ranking activity – (Phase 3)

Content Expert Advisory Panel	
Composition	**Descriptor**
2 Professors in Quality and Safety and leading international experts on teamwork	Experts on teamwork subject matter and quality and safety in healthcare.
1 National Health Service Senior Manager<br>1 Hospital Group CEO<br>1 Hospital Group Director of Human Resources	Senior Healthcare Managers with operational expertise in acute hospital contexts.
2 National experts in teamwork	Individuals who and are renowned for their experiential knowledge and practice in the field and are currently conducting research in the Irish healthcare context
2 Patient Advocates	Service users with knowledge of acute hospital contexts from a user's perspective

Reproduced with permission (15)

Supplementary file 2

Case Study context criteria

**Criteria**	**Case Study 1** *(CS1)*	**Case Study 2** *(CS2)*
Health system context	Ireland	The Pacific Northwest of the United States
Hospital Type	Quaternary academic public teaching hospital (>600 beds)<br><br>All staff are employed by the hospital.	Quaternary academic not-for-profit medical centre (> 450 beds)<br><br>Staff are employed by the hospital with the exception of physicians who contract with the hospital.
Intervention descriptor	To change to a hospital-wide collaborative process of daily *takeover of care* from the on-call GIM team.	To strengthen inter-professional collaborative practice and facilitate practice transformation through development and implementation of structured inter-professional bedside rounds (SIBR).
Primary goal	To ensure care of patients admitted from the Emergency Dept. via the un-scheduled care medical pathway is taken over by the most appropriate medical specialty within 24 hours of admission where possible and that there is a more equitable daily distribution of workloads across all medical specialties to ensure safer and better quality of patient care.	To improve relational co-ordination (team communication and relationships) because of high Registered Nurses (RN) turnover; low patient satisfaction and high re-admission rates for patients.
Intervention driver	Internal- Division of Medicine and hospital management	Internal and external- An academic practice partnership between the School of Nursing and the AHF care team with funding from the Health Services Resources Administration (HRSA).
Leadership Support for the intervention	Leadership support included active participation and attendance of the Chief Executive Officer (CEO), Chief Operations Officer (COO) and Executive Clinical Director (ECD) at meetings and workshops related to the intervention.	Leadership support included attendance during project initiation and close out and at a celebratory workshop.

Team Structure and Composition	The intervention involved formation of a "GIM intervention team"<br><br>Following an invitation from facilitators to the GIM group for representation from each medical specialty, medical consultants self -selected to participate in the project team. The CEO, COO and ECD were considered core team members and the team was facilitated by internal facilitators with expertise in lean methodology and organisational change.	The intervention involved formation of a "change team" comprised of inter-professional front-line care team members and grant team members.<br><br>This purposefully selected change management team comprising multiple disciplines (medical nursing and allied health professionals) from across the advanced heart failure (AHF) faculty and was facilitated by an external research team.
Duration of Intervention	15 months	5 Years
Methodology	The team intervention was underpinned by lean six sigma *Define Measure Analyse Improve Control (DMAIC)* methodology (20) An intensive data collection phase was followed by a workshop to co-design a new way of working and was subsequently followed by a series of monthly meetings and workshops interspersed with smaller stakeholder engagement sessions. A new process was trialled and iterated over three *Plan Do Study Act* cycles. This was followed by a six-month control phase once the intervention was embedded.	Following a grant application process and formation of the change team, one year intensive training in TeamSTEPPs (25) was followed by a longitudinal series of twelve leadership workshops delivered over a three year period on a quarterly basis. Workshop content was tailored to needs identified by the change team and incorporated a shift to Structured Inter-professional Bedside Rounds (SIBR) in the in-patient setting. Purposefully selected workshop topics included: improving work and team processes; communication; relational co-ordination using a variety of evidence-based interventions e.g. TeamSTEPPs, leadership coaching and presentations from field experts
Numbers of interview participants out of total no of team members invited to participate	N= 19/22 (86%)	N= 16/24 (66 %)<br><br>*Only front-line care team member participants were invited to participate in interviews (not members of the research team or TeamSTEPPs trainers)*

Reproduced with permission (15)

Supplementary file 3

Interview format Case study 1

Study Title: *An evaluation of theories on the enablers or barriers to effective multi-disciplinary healthcare team interventions in acute hospital contexts*

Purpose of team intervention: to change the general internal medicine, '*post call takeover of care (TOC)*' process in a tertiary acute hospital context in Ireland

Project team membership:
General Internal Medicine Consultants across the following specialties: Care of the Older Person, Respiratory, Acute Medicine, Neurology/Stroke, Endocrinology, Gastroenterology, Infectious Diseases, Rheumatology; Members of the Transformation Office; Senior Management team including: Head of Strategy; Chief Executive Officer, Chief Operating Officer, Executive Clinical Director and Director of Nursing

Aim of study: to understand how the *General Internal Medicine Takeover of Care* change of process intervention (hereafter called *"the GIM project"*) was implemented and to explore and test our theories about what worked for whom, in what conditions, why, to what extent and how.

Introduce self, explain research aims briefly, check interviewee has read the information sheet, answer any questions, ask interviewee to sign consent form and advise they can keep copy of information and/or consent sheets. Request permission to record - explain processes.

Background questions

- Can you tell me a bit about your professional background / time as an X professional?
- How long have you been in your current role / and how long overall have you worked in this organisation? Can you briefly tell me about your current role?
- Why and how did you become involved in the GIM Project?
- Thinking back to before the project began, can you remember what you (and/or your colleagues) expected from the GIM project?

In what capacity were you involved in the GIM project- (*Probe*: as a facilitator/ participant/ administrator or attendee at meetings)?

Outcomes

1. What was your impression of the GIM project **team** meetings? *What worked well and what didn't work so well? What makes you say this? Why was this the case do you think? How did the meetings lead to that outcome?*
2. *Outside of the regular GIM project meetings meetings, were there any discussions about the GIM project with others on the project team? Probe- In what fora?*
3. Do you feel the GIM Project had an impact on your day to day practice? What makes you say that? Can you give me an example?
4. How did this impact your own specialty team? What makes you say this? Why was this the case do you think? How did the meetings lead to that outcome?
5. Have the GIM project outputs been sustained and continued by the team? Why do you say this? What are the challenges to sustaining this in the team? Or is there anything that has helped sustain it?

Exploring Contexts, Mechanisms and Outcomes

First:

Interrogate for each outcome mentioned previously (new or existing CMOCs):

Questions to elicit outcomes:

Ask participant to reflect on the main outcomes they observed as a result of the GIM project (some outcomes may have been mentioned in answers to questions above).

- Probe: So, we're interested to hear what you perceive the impact of GIM project to have been, for instance, what the outcomes have been for individuals, the project team, your own specialty team and patients.

Questions to elicit mechanisms*:*

- Probe*: We are interested in understanding your experiences of the GIM project and in particular your views on what worked, how and why it worked, and who it worked for. We are also interested in what you think helped to trigger or enable these outcomes. For instance, what do you think changed in people's minds (what were people's reactions and reasoning) that led to those outcomes?*

What changed for people? What triggered that change do you think? Why do you think that outcome was observed?

Questions to elicit contextual conditions*:* What is it about this team or the setting or the sessions that led to that outcome or that fostered/triggered that [mechanism]?

Then

Use teacher-learner method to go through Team Interventions in Acute Hospital contexts IPT with participant, e.g.

We have noticed that…

From team interventions implemented elsewhere, we know that…

There is some published evidence suggesting…

During previous interviews, healthcare staff mentioned…

During team intervention change processes, we observed…

CMOC1 Inter-disciplinary team approach and Flattened hierarchy

Each team member's voice is heard and considered of equal value **(C)**	Understanding of roles, mutual respect, support and value;<br>Self & team efficacy<br>Perception of shared decision making<br>Common purpose **(M)**	Increased job satisfaction<br>Higher levels of competence<br>Better teamwork<br>Lower feelings of emotional exhaustion<br>Breaking down of inter-professional silos<br>More integrated care<br>Connectivity of the team and Camaraderie<br>And<br>More efficient use of time **(O)**

C I understand your team was made up of: Consultants from different medical specialties, staff from the Transformation office and senior management/ … is that the case….? ….
And how were decisions made at team meetings- did everyone have a say? Were these processes inclusive and open do you think?

M What was the impact of this way of working on people? Research suggests that interdisciplinary teams where everybody's voice is heard helps team members understand each other's roles, creates a sense of mutual respect, support and value; self & team efficacy and a perception of shared decision making. Was that something you experienced?

O How do you think this way of working impacted on you and on others in the team?

CMOC2 Effective Communication and Shared Understanding of Goals

(C)	(M)	(O)
There is clear, simple, open, honest and timely communication in an appropriate and inclusive environment with SMART goal setting	Shared understanding and clarity of role and purpose;<br>Self- worth and value;<br>Perceptions of confidence and trust in the Intervention	Positive engagement of the team<br>Situational awareness<br>More integrated planning<br>More efficient use of time<br>And better chance of success

C How would you describe the communication and work of this team towards developing the goals for the GIM project? What was the impact of this way of working?

M Research suggests that using clear, simple and open communication and clear goal setting can impact engagement of healthcare staff. How did you feel this way of working impacted on you and on others in the team?

O What was the effect of the team's having this culture around communication and goal setting when individuals were taking part in the GIM sessions? Did it promote engagement of the team? Were individuals aware of what was going on? Was it an efficient use of time? Do you think it contributed to the success of the project?

CMOC 3 Leadership support and alignment of team goals with organisational goals

There is genuine leadership support in the form of tangible resources and positive acknowledgement of staff<br><br>And alignment of team goals with organisational goals through effective engagement and dialogue **(C)**	Motivates, empowers and engages staff, Enacts a sense of team efficacy; a perception of sense making and a shared sense of responsibility and accountability **(M)**	Team pride and camaraderie; Connectedness and confidence in the broader system; Easier implementation and sustainability of the intervention **(O)**

C Did hospital leadership/management demonstrate their support for the GIM project? If so how? If no, what makes you say this?
What was the level of involvement from leadership? What makes you say this? How would you describe the engagement and dialogue of leadership in the organisation with the GIM project?
Did the GIM project team goals align with organisational goals? Why do you say this?
Was there an acknowledgement of the contribution of team members to the project…in what form?

M What was the effect on the team as a result of receiving this level of/lack of support from leadership in the Mater? Research suggest that it can engage, motivates and empower staff What about enacting a sense of team efficacy; creating a perception of sense making and a shared sense of responsibility and accountability. How did you and on others on the team react to this?

O What impact did this have on the team and/or the project? Did this effect the team's ability to achieve outcomes? Can you give me an example?

CMOC5

Broad and purposeful selection of team composition<br>*With*<br>Physician engagement and support if intervention has a clinical focus **(C)**	Feelings of knowledge confidence and competency<br>Psychological safety<br>And<br>Perception of power and influence **(M)**	Legitimacy of the Intervention<br>Better and timelier "buy in"<br>Staff satisfaction<br>Translation of intervention outcomes to practice and better chance of sustainability **(O)**

C How would you describe the makeup of team members in this GIM project team? Were there any people who should have been there that were not? For this kind of process change, who do you think it was crucial to involve in the GIM/ [NHS Team intervention] meetings?

M There is some published evidence suggesting that having broad team composition and physician engagement in the process enacts feelings of knowledge confidence and competency- was this your experience?

O What impact did this have on the team? Did this effect the team's ability to achieve outcomes? Can you give me an example?

M During previous interviews, healthcare staff mentioned that teams made up of a range of disciplines gives a sense of psychological safety – would you agree? How/ why?

O Did this impact on outcomes? What makes you say this?

M Do you think this influenced attendees' perceptions of power and influence?

O What impact did this have on outcomes?)

CMOC6

If team members have positive personal relationships or prior experience of a positive working relationship and/or an established social network **(C)**	Perceptions of Trust<br>Perceptions of Psychological Safety<br>Shared understanding of experiential knowledge of team: ways of working, skill-sets<br>Likes and dislikes **(M)**	Better engagement in intervention and Easier implementation<br>Ability to progress intervention issues informally<br>Distribution of work according to skill-sets<br>More honest and open communication<br>More integrated planning<br>Quicker recovery from conflicts **(O)**

C How would you describe the interpersonal relationships among team members on the GIM team?

M What was the impact of having/ not these strong (?) relationships among the team when the GIM project was being delivered? In previous interviews with healthcare staff, they indicated that strong interpersonal relationships can enact perceptions of trust, psychological safety. Shared experiential knowledge of ways of working and likes and dislikes of other team members…was that your experience?

O How did this affect the team's performance or how the team worked together? Was there any impact on the outcomes for the project or for the GIM project team and/or the GIM Consultants as a group?

Close out: We're coming to the final couple of questions…

Would you recommend other organisations take part in interventions like the GIM project? Why/why not? How would you describe the GIM project to them?

Was there anything else you wanted to mention today that I haven't specifically asked about?

Thank participant for their time and for sharing their experiences.

Reproduced with permission (15)

Supplementary file 4

Interview format Case study 2

Interview Guide

1. Which leadership workshops did you attend? Provide list of workshops with brief description.

1a. Which ones were the most impactful to you? Least impactful?

1b. How has participation in leadership workshops impacted ***you?*** *Your leadership skills? Your competency/skills in communication, conflict and influencing others?*

Probe: What are the ways in which your ability to communicate effectively have changed because of workshop participation?

Probe: How has your working style evolved over the past several years? How, if at all, do you think this has been influenced by participation in these workshops? As a result of participating in the workshops?

These next questions are about you and your team. Can you describe what your team looks like? Are you referring to a unit? Core group of individuals? What roles are those individuals?

2. What are the ways in which ***you interact*** *with your team/other units? Is it different from before the workshops? In what way?*

Probe: How has your approach to facilitating change and mediating conflict evolved?

Probe: How have you applied concepts/topics that you learned to influence or coach others? Have they been effective?

3. What changes have you seen in ***your team*** *and how they function?*

Probe: Members from your team have attended some of the workshops. In what ways, if at all, how has participating in the workshops or other team processes that occurred as a result of this project, influenced how your team functions?

Probe: Describe some of the changes that you have seen in your team. Do you think the changes are a result of the team training or other activities that happened because of the grant? How is this different from before?

4. From our records, you have been involved with the relational coordination surveys. If you recall, RC looks at relationships and communication. They focus on shared goals, shared knowledge, mutual respect, frequent communication, timely communication, accurate communication, and problem-solving communication. Do any of these stand out as having improved? Or unchanged in your workplace. How useful—if at all-- were these dimensions in your work? What about the other tools we worked with in the workshops (e.g. liberating structures, HROs, conflict, coaching)

*5. From your vantage point, what changes do you see in collaborative practice within UW cardiology? (****systems-level*** *change)*

6. Is there anything else that came out of participating in the leadership workshops that you feel is important for us to know? What was missing?

Reproduced with permission (23)

Supplementary file 5

Detailed worked example of IPT 6 evolving to MRT 10

IPT 6	Context	+ Mechanism	= Outcome	CMOC generated from two sources- systematic search of literature using realist synthesis as well as interviews with key informants
6<br>Personal Relationships	*If*<br>team members have positive personal relationships or prior experience of a positive working relationship and/or an established social network	*Then this enacts:*<br>Perceptions of Trust<br>Perceptions of Psychological Safety<br>Shared understanding of how the team works, skill-sets, likes and dislikes<br>Openness to collaborate	*resulting in:*<br>Better engagement in intervention and Easier implementation<br>Ability to progress intervention issues informally<br>Distribution of work according to skill-sets<br>More honest and open communication<br>More integrated planning<br>Quicker recovery from conflicts	If team members have positive personal relationships or prior experience of a positive working relationship and/or an established social network, then this enacts perceptions of trust and psychological safety; shared understanding of how the team works, skill-sets, likes and dislikes; openness to collaborate; better engagement in intervention and easier implementation; ability to progress intervention issues informally; distribution of work according to skill-sets; more honest and open communication; more integrated planning and quicker recovery from conflicts.

Step 1 Data preparation

The data from the audio files were transcribed *(CS1)* and uploaded *(CS1 and CS2)* to NViVo software (28) Each of the transcripts was read and annotations were made in terms of initial observations relating to the theories.

Annotation example CS1 and/or screenshot	Annotation example CS2
Source : C1F1<br><br>*"Those informal meetings that were happening hadn't been happening, that were happening we'll say a decade ago weren't happening now because everybody has got so busy and the 10 o'clock meeting every morning gives them the opportunity to come and meet and they do a fair amount of business, very quickly afterwards*	Source: C2RN2<br><br>*Can I ask a question? So basically, what I heard you say is that sort of participating in these workshops have helped you develop relationships, right?*

about consults and stuff. So it's been positive from that. I think now they are kind of more open to a change thing, they've been brought through a change of process".<br><br>**Annotation:**<br>Query the process developed more positive working relationships i.e. were more of an outcome/ ripple effect than a contextual enabler in some instances.<br>Will need to query more from the Consultant's perspective in subsequent interviews. For next interviews...should consider that there are possibly two CMOCs generated here relating to Personal Relationships- some relationships were established prior to this intervention and may have been an enabler and others were an un-intended outcome of the intervention and may be of benefit.	*Yes.*<br><br>*And foster those relationships. And then maybe improving a little bit how to provide feedback across the disciplines, right? Or professions?*<br><br>*Yeah.*<br><br>*So do you think it was-- the relationship part, do you think it was because the act of sort of engaging together on a project that you then were working together all the time, and that's what sort of helped develop those relationships? Or are you saying that you develop relationship-building skills that you then implemented to help--? Do you know what I'm saying, the difference, right?*<br><br>**Annotation:** The importance of inter-personal relationships is likely to be very strong in CS2 because of the specific focus of the intervention on relational co—ordination.

**Step 2 CMOC extraction and elicitation**<br>Using deductive reasoning and inductive reasoning - Data were coded to adult nodes 6.0 and or new child note related to adult node. Child nodes were named. CMOCs were extracted and/or new CMOC elicited

CS1	CS2
**Sample CMOC extraction**<br>Source: C1P12<br>*Participant: Yeah I suppose, I think when it started and people could see the benefits of it, like I say to you that's really where they allowed the momentum to be maintained and not accelerated and people sort of bought into this. I don't think it will ever go back to the way it was and I think when people saw that the fringe benefits of this outweighed the negatives, that's what allowed it to for the change to be implemented into stake, I'm not sure if that's answering your question!*<br><br>***Interviewer: Well it's looking at what has happened and then how do you explain that so you are talking about the consequences and I'm trying to work back and see how did you arrive at those, why did that happen.***<br><br>*Participant: Yeah .. again I suppose how it happened is and why it is the way it is because like anything if you get a sense that something is successful in any small way then that will accelerate, that's almost starting in the middle of the story.*	**Sample CMOC extraction**<br>Source: C2ARNP2<br>*"And then I also think just getting to-- having the workshops with the nurses, and Teletech, and then getting to know them more on a social level. I think there's a lot more interaction going both ways. Them feeling free to ask me more questions, or me feeling free to ask things of them or ask them questions, because they're-- when you go into a workshop with somebody, especially when we had the, what was it, the team steps where we were practicing SIBR, and I made them all say scenarios and say certain things, then you get to know people more on a personal level. And we also did little introductions at each one of the workshops. Tell me who you are, and where you work, and one thing about you or something like that. And so getting to know people on a personal level makes it a lot easier to work with people as a group, because you're like, "Oh yeah, hey [Name of RN], I remember you."*<br><br>*Yeah, your guards are less up and, yeah, I think it's just much more cool. And then, now that they're joining us in rounds every day, I know who they are, what to expect of them, they know what to expect of me. So I think there's a big improvement there. Since the nurse practitioners I work with didn't really attend the workshops, I don't know that my relationship necessarily has, just from my side, has changed as far as what I said before, being, listening to them before reacting kind of thing. But I think that's a little bit of a downfall.*
**C** A sense of success of the intervention bred a feel good factor among the group and<br><br>**M** Enacted a perception that this was something to be part of; creating legitimacy for the intervention; and sense making i.e. that the positive outputs out-weighed the negative.<br><br>**O** As a result, change was implemented with the likelihood of never going back to old way i.e. the intervention is likely to be sustained	**C** Team members who attended the workshops got to know each other on a more personal level and this facilitated<br><br>**M** Trust; Perceptions of Psychological Safety ; Awareness of expectations of each other<br><br>**O** Better inter-professional relationships and easier work practices

**Step 3 Using CMOCs to refine IPT 6**<br>Using retroduction- The respective narrative was analysed under each adult or child node. CMOCs were reviewed to determine how they aligned with the original IPT.	
**CS2 Decision making tree example**<br><br>**CMOC** As above Source: C1P12<br><br>**Support/Refute/Refine –** Support for Ripple 6.1.1<br>**How decision?**<br>Inference that once there was a "feel good factor" among the physicians as a result of the initial perceived success, then this caused more to happen.<br><br>**Links/ Ripples /Effects**<br>Likely to be a platform on which to build further change...success breeds success breeds success**-** a self -perpetuating theory that could be taken further with the right intervention.<br><br>**Notes**<br>Perhaps this ripple theory should be called "feel good factor among the team"...this might be more important than IPT 6 where the emphasis is on "prior working relationships".<br><br>**Other codes**<br>**IPT 4a** ripple theory which was not ranked for testing- If there is a strong recurring pattern may need to re-consider as an enabler. (Discuss with EMcA and AD).	**CS2 Decision making tree example**<br><br>**CMOC** As above Source: C2ARNP2<br><br>**Support/ Refute/ Refine -**Refine<br>Not a prior working relationships but a newly developed relationship as part of the intervention so refine.<br><br>**How Decision?**<br>Narrative is explicit<br><br>**Links/Ripples/Effects**<br>Better camaraderie perhaps<br><br>**Notes**<br>The fact that some staff did not get to attend was a disadvantage. This is mirrored in other narratives with the second year fellows came in mid intervention. Discussion point for sustainability.<br><br>**Other codes**<br>IPT 1 and IPT 5

Step 4a Collating evidence -Each of the memos specific to the five individual programme theories was read thoroughly and patterns of regularity across files were noted. Thought processes were annotated. Decisions were logged. Supporting evidence for thought processes was logged with examples of supporting quotes.

Case Study 1

6.1Thought process

I. IPT 6.0 is supported

Supporting quote

C1SM2

Interviewer: *"Ok, yeah, so is that about building relationships with them do you think"?*

Participant: "*Yeah definitely like, I certainly would have built relationships with the consultants and when you're trying to implement any change having support at that level with the consultant on board is huge and we've definitely built relationships with those consultants and are able to approach them now in a more casual manner I suppose and they have an understanding of what we're trying to do and achieve and they would give us time".*

Decision: IPT 6.0 needs to be dis-aggregated in terms of Cs, Ms and Os for clarity and also seems to be a ripple effect of good relationships getting better. Split next iteration of IPT 6 into two: 6.1.1 and 6.1.2 to reflect this.

Supporting Evidence for 6.1.1

C1F1, C1SM2, C1P3, C1P13

And

6.1.2 Thought process Query ripple

II. It seems most agreed that there was a strong positive culture in the hospital prior to the creation of a forum for consultants to come together to work on this intervention even where there were some tensions between two specialties. A number talked about the success of this intervention and it being a platform on which to build future work; the camaraderie thereafter and good relationships getting even better. Many spoke about the success or sense of satisfaction or achievement that it

Case Study 2

6.1 Thought process

i. Narrative supports IPT6 across 4 interviews *as is* however, there is no evidence to support IPT 6 in others- This is surprising because there is such a focus on relational co-ordination. Perhaps it is implicit in the intervention and therefore not spoken about as much at interviews?

ii. Source: C1P3 - I think refinement is required- having **prior working relationships** in this case study did not seem to matter as attendance at the TeamSTEPPs training together and/or the workshops **created the opportunity for relationship building** and having these opportunities is the enabling piece. As per Source 7 the fact that some people did not get to attend the workshops meant that relationship building did not happen and subsequently for this physician this is perceived as "a downfall".

C1P3

"Then I also think just getting to-- having the workshops with the nurses, and TeleTech's, and then getting to know them more on a social level. I think there's a lot more interaction going both ways. Them feeling free to ask me more questions, or me feeling free to ask things of them or ask them questions, because they're-- when you go into a workshop with somebody, especially when we had the, what was it, the TeamSTEPPs where we were practicing SIBR, and I made them all say scenarios and say certain things, then you get to know people more on a personal level. And we also did little introductions at each one of the workshops. Tell me who you are, and where you work, and one thing about you or something like that. And so getting to know people on a personal level makes it a lot easier to work with people as a group, because you're like, "Oh yeah, hey Nika, I remember you.

"Yeah, your guards are less up and, yeah, I think it's just much more cool. And then, now that they're joining us in rounds every day, I know who they are, what to expect of them, they know what to expect of me. So I think there's a big improvement there. Since the nurse practitioners I work with didn't really attend the workshops, I don't know that my relationship necessarily has, just from my side, has changed as far as what I said before, being, listening to them before reacting kind of thing. But I think that's a little bit of a downfall".

had worked and there appeared to be a sense of personal contribution especially amongst the facilitators- a real sense of team efficacy, a sense of being part of something positive which was good for morale.

Decision treat this as a ripple theory: Ripple PT 6.1.2 Good relationships even better.

Supporting evidence for 6.1.2 (Ripple theory)
C1F1, C1SM1, C1P1, C1SM2, C1SM3, C1P7, C1P13, C1F3

Supporting quote

iii. Thought process…need to mine the data again…looking for **opportunities to meet and relationships to develop**.

iv. Thought process C2RN2 -The interviewer makes an important distinction and “helps unpack the black box”! Questions what the enabling factor was i.e. the team members working together all the time or whether it was learning new skills in relationship building that helped. Interviewee says former because it appears to build mutual understanding, mutual appreciation and understanding of roles broadening perspectives, greater appreciation of each other’s worlds.

Opportunities for relationship building again is the enabling factor- being in room together

C2RN2

“So do you think it was-- the relationship part, do you think it was because the act of sort of engaging together on a project that you then were working together all the time, and that's what sort of helped develop those relationships? Or are you saying that you develop relationship-building skills that you then implemented to help--? Do you know what I'm saying, the difference, right?”

“It was probably the first. I think it was just spending the time”.

Okay.

“But I think part of spending the time has helped me have a better insight into the world of a cardiologist attending in at UWMC, and sort of how to engage with that. Sometimes the nurses are like, "The resident didn't put an order-- they didn't discontinue the chest tube order. There's no chest tubes at 2:00 AM." And I'm like, "Okay, so I'm not going to follow up on that [laughter]." So I think it's sometimes that we're like, "This is important in my world." And so, I think that's helped me have greater appreciation for their world, and then how do we engage in that. Hopefully in a meaningful way. Yeah”.

v. Source 16-Inter-personal relationship development is hard in hospital contexts because of the amount of staff turnover. Source 16. This is something that might be relevant on discussion section and ties into

	transitioning piece in team composition PT5, effective communication PT2. **Getting to know you** is really important for foundational work….needs to be factored in when on-boarding new staff. In a busy clinical world, this might be challenging but at the same time necessary. **Creating the opportunities for social networking on a formal basis** like the case conference helps collaborative practice…query it also has an impact on relationship building.<br><br>*C1P11*<br>*"And the longer you work with someone, the more you're able to know their style of communication and things like that. It has improved. I'm going to say overall it has improved. But it has been a difficult road because of the massive amount of turnover".*<br><br>Inter-dependency- PT 2 Positive personal relationships allow for more effective communication because of increased opportunities for conversations (especially informal ones) to happen.<br><br>Decision- Support with some refinement in terms of **creating opportunities for inter-personal relationships to develop**.<br><br>***Supporting evidence***<br>C2RN2, C"ARNP2, C2RN4, C2RN7<br>**Supporting quote**<br><br>*C1P4*<br>*"I think like anything relationship-based, we do really well at on [Name of ward/ area Five Southeast]. Providers interact with each other. I mean, even just yesterday I was walking down the hall and you see a provider sitting with a nurse talking about what's going on with their lives outside of work. Which really just creates a space where you feel more comfortable speaking up. You bring up things that you wouldn't with a provider that you didn't have a relationship with. And you have the same goals because you know each other as people and not just providers. So I think that's probably one area."*

**Step 4b Refinement verification**- a randomly chosen sub sample of four files was double coded by another member of the research team AD to challenge the primary researcher's assumptions and interpretations thereby adding rigour and robustness to the process (32).	
Refinement verification for IPT 6 from double coding by ADB<br>Thought process re: CMOC as follows.<br>**IPT 6- Personal Relationships**<br>**Supported**<br>**Context**<br>- Confirmed from IPT, nothing major additional. However, interesting that people alluded to this in other ways (time to speak to colleagues, give and take) and didn't necessarily state good working relationships but talked around it. Do participants underestimate value of this?<br>**Mechanisms**<br>- Trust, psychological safety.<br>**Outcomes**<br>- Burden sharing mentioned a couple of times. Addressing concerns/issues (conflict resolution). More effective planning.<br><br>**Decision required:** Discussed with ADB how I have split PT and added in a ripple PT…is in agreement.	***Refinement verification for IPT 6 from double coding by ADB***<br>Thought process re: CMOC as follows.<br>**IPT6 Personal relationships**<br>**Very strong, strongly supported**<br>**Context:**<br>Protected time for team training / for team to meeting formally or informally – this is key and coming out very strongly as a key driver of intervention effectiveness. "I think it was just spending the time…..interacting and communicating" C2RN2<br>Increasing familiarly, less formality "*People are more friendly." C2AHP1*<br><br>*"We also did little introductions at each one of the workshops. Tell me who you are, and where you work, and one thing about you or something like that. And so getting to know people on a personal level makes it a lot easier to work with people as a group, because you're like, "Oh yeah, hey [Name], I remember you." C2ARNP2*<br><br>*I think it is very important at the beginning to just even-- talking about these relationship coordination issues, and start the dialogue. C2AHP1*<br><br>**Mechanisms:**<br>understanding of pressures on other professionals, greater appreciation, empathy<br>*But I think part of spending the time has helped me have a better insight into the world of a cardiologist attending in at UWMC, and sort of how to engage with that. Sometimes the nurses are like, "The resident didn't put an order-- they didn't discontinue the chest tube order. There's no chest tubes at 2:00 AM." And I'm like, "Okay, so I'm not going to follow up on that [laughter]." So I think it's sometimes that we're like, "This is important in my world." And so, I think that's helped me have greater appreciation for their world, and then how do we engage in that. Hopefully in a meaningful way. C2RN2*<br><br>*I think I know to step back and think about a situation first and then look at it from all angles and think about, and then really listen to the other person's story or what they're really trying to tell me or trying to get out, even though that's not what they're conveying. Sometimes reading between the lines if they're really emotional about something. C2ARNP2*

	Collective mind-set, empowerment<br>*Well, I haven't really done that either but let's go over and look at my patient's chart who had that," and, "Let me help you." I think there's more of that. We're all our own-- we're all a group. Not just, "I'm going to survive my day."*<br>*C2RN2*<br><br>*So I feel like there's that much more of, how can we collaborate together for the best possible outcome.C2AHP1*<br><br>*But if you can make people like they're part of something bigger than some of these smaller subsections, they feel more empowered. C2ARNP1*<br><br>**Outcomes:**<br>Positivity, team morale, positive working environment<br>I think there's a lot of positivity around that team and that interaction because of that. That has been great. C2RN2<br><br>Improved communication, ease of communication, through building interpersonal relationships<br>*"I think they've probably all improved at some level for everyone because I think people are communicating more. I think the communication is more value-added. I think there's a couple physicians in particular, when we started, who had very poor relationships with the nurses, who now have very strong relationships with the nurses, and I think feel very differently about their time on the unit. C2RN2*<br><br>*I think I'm more-- it's just allowed, I think, better personal-- like just, you know my name, you kind of know where I'm at, you know what I'm working for, and allow me to reach out to people a little bit more comfortably" C2RN2*<br><br>Proactive helping behaviours<br>*"Well, I haven't really done that either but let's go over and look at my patient's chart who had that," and, "Let me help you." I think there's more of that. We're all our own-- we're all a group. Not just, "I'm going to survive my day." C2RN2*<br><br>*there's a couple of attendings who, when they're on rounds, actively come and talk to me about, "Can you help me with this?" or "This was an issue," or "This went really well," and just, I think, see-- it's helped me gain with some of the Attendings that I'm seen more as a*

	partner when they're on rounds with them, and then someone who manages the unit, like someone who can really help the team and help the patients. So I think that has been a real positive<br>*C2RN2*<br><br>**Supporting quote**<br>*"It's really all about relationship building… Which sounds hokey, but boy is it true."*<br>*C2ARNP2*<br><br>**Decision**- Strongly supported. Refine to include more detail of mechanisms and outcomes as illustrated.

Collating evidence- Demi-regularities within CS1

Decision IPT 6 supported and dis-aggregated to 6.1.1 and 6.1.2 (ripple)

C1 Positive personal relationships<br>C2 Prior shared working experience<br>C3 Established social network	M1 Trust<br>M2 Psychological Safety<br>M3 Shared understanding of how the team works, skill-sets, likes and dislikes<br>M4 Openness to collaborate	O1 Better engagement in intervention<br>O2 Easier implementation<br>O3Ability to progress intervention issues informally<br>O4 Distribution of work according to skill-sets<br>O5More honest and open communication<br>O6More integrated planning<br>O7Quicker recovery from conflict	**CS1 Split PT 6.1.1**<br>If team members have positive personal relationships or prior experience of a positive working relationship and/or an established social network, then this enacts perceptions of trust and psychological safety; Shared understanding of how the team works, skill-sets, likes and dislikes; openness to collaborate and results in: better engagement in intervention and easier implementation; ability to progress intervention issues informally; distribution of work according to skill-sets; more honest and open communication; more integrated planning and quicker recovery from conflicts.
C1 Positive personal relationships<br>C2 Prior shared working experience	M1 Trust<br>M2 Psychological Safety	O1 Better engagement in intervention<br>O2 Easier implementation	**CS1 Split PT 6.1.2 (Ripple)**

C3 Established social network	M3 Shared understanding of how the team works, skill-sets, likes and dislikes<br>M4 Openness to collaborate	O3Ability to progress intervention issues informally<br>O4 Distribution of work according to skill-sets and **burden sharing**<br>O5More honest and open communication<br>O6More integrated planning<br>**O7Quicker recovery from conflict**	If team members have positive personal relationships or prior experience of a positive working relationship and/or an established social network, then this enacts perceptions of trust and psychological safety; shared understanding of experiential knowledge of team: ways of working, skill-sets; likes and dislikes; better engagement in intervention and easier implementation; ability to progress intervention issues informally; distribution of work according to skill-sets and **burden sharing**; more honest and open communication; more integrated planning and **quicker recovery from conflicts.**
**CS1 Demi-regularities -Refinement verification (after double coding by ADB)**<br>Decision 6.1.1 supported (=6.2.1) and 6.1.2 Refined to 6.2.2			
C1 Positive personal relationships<br>C2 Prior shared working experience<br>C3 Established social network	M1 Trust<br>M2 Psychological Safety<br>M3 Shared understanding of how the team works, skill-sets, likes and dislikes<br>M4 Openness to collaborate	O1 Better engagement in intervention<br>O2 Easier implementation<br>O3Ability to progress intervention issues informally<br>O4 Distribution of work according to skill-sets<br>O5More honest and open communication<br>O6More integrated planning<br>O7Quicker recovery from conflict	**CS1 Split**<br>**PT 6.2.1 unchanged from 6.1.1**<br>If team members have positive personal relationships or prior experience of a positive working relationship and/or an established social network, then this enacts perceptions of trust and psychological safety; Shared understanding of how the team works, skill-sets, likes and dislikes; Openness to collaborate better engagement in intervention and easier implementation; ability to progress intervention issues informally; distribution of work

			according to skill-sets; more honest and open communication; more integrated planning and quicker recovery from conflicts.
**C1 Experience of success of the intervention**<br>**C2 Experience of positive working relationships**	M1 Legitimacy of the intervention<br>M2 Perception of sense making<br>**M3 A sense of personal contribution and connectedness with something positive**	O1 Need for team members to align with this success<br>**O2 Boost to team morale/ Feel good factor**<br>**O4 A sense of camaraderie**<br>**O2 Sustainability of the intervention**<br>**O4 Perception of being a platform on which to build future work**	**CS1 Split Ripple PT 6.2.2**<br>If **there is experience of success of an intervention** and team members **experience positive working relationships within a positive culture and atmosphere**, this enacts a **sense of personal contribution, a sense of connectedness with something positive** resulting in a need for **team members to align with this success; a boost to team morale; a sense of good relationships getting even better; a sense of camaraderie; sustainability of the intervention and a perception that the success is a platform on which to build future work.**
**CS1 Demi-regularities- Resource and reasoning mechanisms dis-aggregated**<br>Decision: minor refinements to reflect the disaggregation 6.3.1 and 6.3.2			
**Context**<br>Positive personal relationships<br>Shared working experience<br>Established social network	**Mechanisms**<br>**Resource**<br>Skilled facilitation to capitalise on positive personal relationships<br>**Reasoning**<br>Trust<br>Psychological Safety<br>Shared understanding of how the team works, skill-sets, likes and dislikes<br>Openness to collaborate	**Outcomes**<br>Ability to progress intervention issues informally<br>More honest and open communication<br>Burden sharing<br>Distribution of work according to skill-sets<br>Conflict resolution and quicker recovery from conflict	**CS1 Split PT 6.3.1**<br>If team members have positive personal relationships/ shared work experiences or an established social network **and skilled facilitators capitalise on these to progress issues**, then this enacts perceptions of trust and psychological safety; Shared understanding of how the team works, skill-sets, likes and dislikes, openness to collaborate and results in: ability to progress

			intervention issues informally; more honest and open communication; burden sharing; distribution of work according to skill-sets; conflict resolution and quicker recovery from conflicts.
**Context**<br>Experience of positive working relationships and success of the intervention	**Mechanisms**<br>**Resource**<br>Demonstration and acknowledgement of success<br>**Reasoning**<br>A sense of personal contribution<br>Connection with something positive	**Outcomes**<br>Team members aligning with this success<br>Boost to team morale/ Feel good factor<br>Evidence of camaraderie<br>Sustainability of the intervention<br>Perception of being a platform on which to build future work	**CS1 Ripple PT 6.3.2**<br>If there is **demonstration and acknowledgement of intervention success in a context of positive working relationships**, this enacts a sense of personal contribution and connection with something positive resulting team members aligning with this success; a boost to team morale, **(a feel good factor)** and evidence of camaraderie; sustainability of the intervention and a perception that the success is a platform on which to build future work.

**Collating evidence: Demi-regularities within CS2**<br>Decision IPT 6 supported with refinement and dis-aggregation of resource and reasoning mechanisms			
C1 Positive personal relationships<br>C2 Shared working experiences<br>C3 Established social network	**Mechanisms**<br>**Resource**<br>Workshops, SIBRs,	O1 More collaborative practice<br>O2 Ability to progress intervention issues informally	**CS2 PT 6.1**<br>**When there are opportunities for positive personal relationships to develop e.g. during team**

	**Reasoning**<br>M1 Trust<br>M2 Psychological Safety<br>M3 Shared understanding and experiential knowledge of team: ways of working, communicating<br>M4 Broadened perspectives	O3 Distribution of work according to skill-sets<br>O4 More honest and open communication<br>O5 More integrated planning	**training/SIBRs/shared work experiences or** if there is an already established social network where positive personal relationships have developed, this enacts feelings of trust, psychological safety, shared understanding; experiential knowledge of team, ways of working and communicating and broadening of perspectives and results in: more collaborative practice, ability to progress intervention issues informally, distribution of work according to skill-sets, more honest and open communication and more integrated planning.
**CS2 Demi-regularities -Refinement verification (after double coding by ADB)**<br>Decision: Strongly supported with refinement of detail			
**Contexts**<br>Positive personal relationships<br>Increased familiarity<br>Less formality	**Mechanisms**<br>**Resource**<br>Protected time for team training / opportunities created for team to meet formally or informally<br>Shared experiences via<br>team learning events and meetings<br>**Reasoning**<br>Understanding of pressures on other professionals, greater appreciation & empathy<br>Collective mindset Empowerment<br>Psychological Safety<br>Shared understanding of individuals' skills and attributes and potential to contribute	**Outcomes**<br>Positive team morale and working environment<br>Ease of communication because of inter-personal relationships<br>Ability to progress intervention issues informally<br>Openness and honesty<br>Pro-active helping behaviours<br>Recognition of skill-sets and preferences	**CS2 PT 6.2** If there are positive personal relationships, **increased familiarity and less formality and/or protected time/ opportunities for team to meet formally/ informally to develop personal relationships through shared experiences e.g. team learning events and team meetings,** this enacts **empathy and understanding of pressures on other professionals; a collective mindset; empowerment psychological safety and shared understanding of individuals' skills and attributes and potential to contribute** resulting in **positive team morale and working environment,**

			**ease of communication, openness and honesty, ability to progress intervention issues informally pro-active helping behaviours, recognition of skill-sets and preferences.**
**CS2 Demi-regularities -Refinement (after Zoom meeting with research teams in US)**<br>Decision: dis-aggregate team and organisational context supported minor refinement only			
**Organisational context**<br>Foundation to establish good team relationships had been built by introduction of TeamSTEPPs to 23 units across the hospital.<br>**Intervention/Team Context**<br>Poor relational co-ordination initially.<br>Through the intervention and work done in year 1 & 2 a new context was created that supported the development of positive personal relationships.<br>There was increased familiarity and less formality among team members.	**Mechanisms**<br>**Resource**<br>**Protected time for team training /opportunities created for team to meet formally or informally e.g. shared experiences via team learning events and meetings to support development of these relationships**<br>**Reasoning**<br>**Greater appreciation** and empathy of pressures on other disciplines<br>Shared understanding of individuals' skills and attributes and potential to contribute<br>Collective mindset Empowerment<br>Psychological Safety	**Outcomes**<br>Positive team morale and working environment<br>Ease of communication because of inter-personal relationships<br>Ability to progress intervention issues informally<br>Openness and honesty<br>Pro-active helping behaviours<br>Explicit statement of skill-sets and preferences	**CS PT 6.3**<br>*If*<br>there are positive personal relationships, familiarity and less formality<br>*And the intervention offers*<br>**protected time and/or opportunities for the team to meet formally or informally to support development of these relationships,**<br>*This enacts*<br>**greater appreciation** and empathy of pressures on other team members, shared understanding of individuals' skills and potential to contribute, creates a collective mindset, empowers and gives a sense of psychological safety<br>*And results in*<br>positive team morale and working environment, ease of communication, openness and honesty, ability to progress intervention issues informally

			pro-active helping behaviours and explicit statement of skill-sets and preferences.
**Step 5 Synthesis across studies for MRTs** This final phase of analysis involved a search for demi-regularities (semi-predictable patterns) across the case studies.			
**CS1 Synthesis across studies for demi-regularities**<br>Patterns of regularity across cases studies identified using retroductive processes			
**Context**<br>Positive personal relationships<br>Shared working experience<br>Established social network	**Mechanisms**<br>**Resource**<br>Skilled facilitation to capitalise on positive personal relationships<br>**Reasoning**<br>Trust<br>Psychological Safety<br>Shared understanding and knowledge of team: ways of working; team skills; likes and dislikes	**Outcomes**<br>Ability to progress intervention issues informally<br>More honest and open communication<br>Burden sharing<br>Distribution of work according to skill-sets<br>Conflict resolution and quicker recovery from conflict	**CS1 Split PT 6.3.1**<br>*If*<br>team members have positive personal relationships/ shared work experiences or an established social network and<br>*skilled facilitators*<br>capitalise on these to progress issues,<br>*then this enacts*<br>perceptions of trust and psychological safety; shared understanding, knowledge of team: ways of working, skill-sets; likes and dislikes *and results in*<br>ability to progress intervention issues informally; more honest and open communication; burden sharing; distribution of work according to skill-sets; conflict resolution and quicker recovery from conflicts.
Decision – This ripple PT links with IPT 4a which has come back into play			
**Context**<br>Experience of positive working relationships and success of the intervention	**Mechanisms**<br>**Resource**	**Outcomes**<br>Evidence of camaraderie	**CS1 PT 6.3.2 Ripple**<br>*If*

	Demonstration and acknowledgement of success<br>**Reasoning**<br>A sense of personal contribution<br>Connection with something positive<br>Team members aligning with this success<br>Boost to team morale/ Feel good factor	Sustainability of the intervention<br>Perception of being a platform on which to build future work	There is experience of positive working relationships and success of the intervention<br>*And the interventions allows for*<br>Demonstration and acknowledgement of this success<br>*this enacts*<br>a sense of personal contribution and connection with something positive<br>*And results in*<br>team members aligning with this success; a boost to team morale, a feel good factor and evidence of camaraderie; sustainability of the intervention and a perception that the success is a platform on which to build future work.
**CS2 Synthesis across studies for demi-regularities**<br>Patterns of regularity across cases studies identified using retroductive processes			
**Organisational context**<br>Foundation to establish good team relationships had been built by introduction of TeamSTEPPs to 23 units across the hospital.<br>**Intervention/Team Context**<br>Poor relational co-ordination initially.<br>Through the intervention and work done in year 1 & 2 a new context was created that supported the development of positive personal relationships.<br>There was increased familiarity and less formality among team members.	**Mechanisms**<br>**Resource**<br>Protected time for team training /opportunities created for team to meet formally or informally e.g. shared experiences via team learning events and meetings<br>**Reasoning**<br>Greater appreciation and empathy of pressures on other disciplines<br>Shared understanding of individuals' skills and attributes and potential to contribute<br>Collective mindset<br>Empowerment	**Outcomes**<br>Positive team morale and working environment<br>Ease of communication because of inter-personal relationships<br>Ability to progress intervention issues informally<br>Openness and honesty<br>Explicit statement of skill-sets and preferences<br>Some pro-active helping behaviours, (not for all in CS1)	**PT 6.1 CS2**<br>*If*<br>there are positive personal relationships, familiarity and less formality<br>*And the intervention offers*<br>protected time and/or opportunities for the team to meet formally or informally to support development of these relationships,<br>*This enacts*<br>greater appreciation and empathy of pressures on other team members, shared understanding of individuals' skills and potential to contribute,

	Psychological Safety		creates a collective mindset,<br>empowers and gives a sense of psychological safety<br>*And results in*<br>positive team morale and working environment, ease of communication, openness and honesty, ability to progress intervention issues informally<br>pro-active helping behaviours and explicit statement of skill-sets and preferences.
**Synthesised findings from across CS1 and CS2**<br>Decision: MRT for presentation to research team by UC			
**Intervention/Team Context**	**Mechanisms**	**Outcomes**	**Proposed MRT 10**
Context supports the development of positive personal relationships.<br>There is increased familiarity and less formality among team members.	**Resource**<br>Protected time /opportunities created for team to meet formally or informally to support development of personal relationships<br>**Reasoning**<br>Greater appreciation and empathy of pressures on other disciplines<br>Shared understanding and knowledge of team skills and potential to contribute<br>Collective mindset<br>Empowerment<br>Psychological Safety	Positive team morale and working environment<br>Ease of communication<br>Openness and honesty<br>Ability to progress intervention issues informally<br>Explicit statement of skill-sets and preferences<br>Pro-active helping behaviours /Burden sharing<br>Conflict resolution and quicker recovery from conflict	*If*<br>there are positive personal relationships, familiarity and less formality through prior shared experiences<br>and/or<br>*the intervention offers*<br>protected time and/or opportunities for the team to meet formally or informally to support development of these relationships,<br>*This enacts*<br>greater appreciation and empathy of pressures on other team members, shared understanding of individuals' skills and potential to contribute, creates a collective mindset, empowers and gives a sense of psychological safety

			And results in<br>positive team morale and working environment, ease of communication, openness and honesty, ability to progress intervention issues informally<br>pro-active helping behaviours/ burden sharing, explicit statement of skill-sets and preferences conflict resolution and quicker recovery form conflicts.
**Final MRT 10 Supporting Development of Inter-personal relationships**<br>Decision: Post refinement MRT agreed by research team			
**Team Context**<br>Readiness and openness to an improvement culture<br>**Ripple- New context develops as intervention progresses**<br>This context supports the development of positive personal relationships.<br>There is increased familiarity and less formality among team members.	**Mechanisms**<br>**Resource**<br>Protected time and opportunities for team to meet formally or informally<br>**New inter-personal relationships develop and old relationships are further supported. This has a ripple effect creating a new context.**<br>**Reasoning**<br>Greater appreciation of and empathy for pressures on other disciplines<br>Shared understanding and knowledge of team skills and potential to contribute<br>Collective mindset<br>Empowerment<br>Psychological Safety	**Outcomes**<br>Positive team morale and working environment<br>Ease of communication<br>Openness and honesty<br>Ability to progress intervention issues informally<br>Explicit statement of skill-sets and preferences<br>Pro-active helping behaviours /Burden sharing<br>Conflict resolution and quicker recovery from conflict	**Ripple MRT 10**<br>***If***<br>**There is readiness and openness to an improvement culture**<br>*And the intervention offers*<br>protected time and opportunities for the team to meet formally or informally. **Over time, a new context evolves which supports the development of positive inter-personal relationships** where there is increased familiarity and less formality among team members<br>*This enacts*<br>greater appreciation of and empathy for pressures on other team members, shared understanding of individuals' skills and potential to contribute, a collective mindset, empowers and gives a sense of psychological safety

			And results in<br>positive team morale and working environment, ease of communication, openness and honesty, ability to progress intervention issues informally, pro-active helping behaviours/ burden sharing, explicit statement of skill-sets and preferences, conflict resolution and quicker recovery from conflicts.
Decision - collapse the ripple CS1 6.3.2 evolved from IPT 6 with Ripple IPT4a and evolve into MRT 11 Celebration of Success			
**Context**<br>Experience of positive working relationships and success of the intervention	**Mechanisms**<br>**Resource**<br>Demonstration and acknowledgement of success<br>**Reasoning**<br>A sense of personal contribution<br>Connection with something positive<br>Team members aligning with this success<br>Boost to team morale/ Feel good factor	**Outcomes**<br>Evidence of camaraderie<br>Sustainability of the intervention<br>Perception of being a platform on which to build future work	**Ripple CS1 6.3.2**<br>*If*<br>There is experience of positive working relationships and success of the intervention<br>*And the interventions allows for*<br>Demonstration and acknowledgement of this success<br>*this enacts*<br>a sense of personal contribution and connection with something positive<br>*And results in*<br>Team members aligning with this success; a boost to team morale, a feel good factor and evidence of camaraderie; sustainability of the intervention and a perception that the success is a platform on which to build future work.

Final Ripple MRT 11- Celebration of success

Team Context	Mechanisms	Outcomes	Ripple MRT 11
Experience and acknowledgement of success of the intervention<br>And experience of more positive working relationships	**Resource**<br>Demonstration and acknowledgement of success Dissemination of success story<br>**Reasoning**<br>A sense of personal contribution<br>Connection with something positive<br>Team members aligning with this success<br>Boost to team morale/ Feel good factor	Evidence of camaraderie<br>A new way of working for the team<br>Externally perceived credibility in the intervention and subsequent buy in of other staff<br>New intervention is embedded and sustained<br>Participants demonstrate willingness to engage in other interventions	If<br>There is experience and acknowledgment of success of the intervention and more positive working relationships<br>And the interventions allows for Demonstration and acknowledgement of this success story<br>this enacts<br>A sense of personal contribution, connection with something positive, team members aligning with this success; a boost to team morale/ a feel good factor<br>And results in<br>Evidence of camaraderie; a new way of working for the team, new staff adapting to this as part of the culture; externally perceived credibility in the intervention and subsequent buy in of other staff, sustainability of the intervention and potential to spread, participants willingness to engage in other interventions
